# Dual novel mutations in *SLC26A2* in two siblings with multiple epiphyseal dysplasia 4 from a Chinese family: a case report

**DOI:** 10.1186/s12881-018-0596-7

**Published:** 2018-05-03

**Authors:** Taifeng Zhou, Yongqian Wang, Hang Zhou, Zhiheng Liao, Bo Gao, Deying Su, Shuhui Zheng, Caixia Xu, Peiqiang Su

**Affiliations:** 1 Department of Orthopaedic Surgery, First Affiliated Hospital of Sun Yat-sen University, No.58 Zhongshan 2nd Road, Yuexiu District, Guangzhou, 510080 China; 2 Guangdong Provincial Key Laboratory of Orthopedics and Traumatology, First Affiliated Hospital of Sun Yat-sen University, Guangzhou, 510080 China; 3 Department of Musculoskeletal Oncology, First Affiliated Hospital of Sun Yat-sen University, Guangzhou, 510080 China; 4 Department of Spine Surgery, Sun Yat-sen Memorial Hospital, Guangzhou, 510120 China; 5 Research Centre for Translational Medicine, First Affiliated Hospital of Sun Yat-sen University, No. 58 Zhongshan 2nd Road, Yuexiu District, Guangzhou, 510080 China

**Keywords:** Multiple epiphyseal dysplasia, *SLC26A2*, Targeted next-generation sequence, Compound heterozygote

## Abstract

**Background:**

Multiple epiphyseal dysplasia (MED) is a heterogeneous genetic condition characterized by variable phenotypes, such as short stature (mild to moderate), joint deformities, abnormal gait, scoliosis, and brachydactyly. Recessive mutations in the *SLC26A2* gene cause a phenotype of multiple epiphyseal dysplasia-4 (MED-4). In the present study, we identified novel compound heterozygous mutations in the *SLC26A2* gene in a Chinese family with two affected sibs with MED-4.

**Case presentation:**

Radiographs revealed hip dysplasia, brachydactyly and scoliosis in patient 1. Radiological examinations in patient 2 also showed hip dysplasia recently. Both of them were diagnosed with MED-4. *SLC26A2* c.824 T > C and *SLC26A2* c.1198C > T were identified in two siblings in this family, which were inherited from both parents, one mutation from each.

**Conclusions:**

This is the first Chinese MED-4 family attributed to *SLC26A2* mutations, and these results show that these novel compound heterozygous mutations in *SLC26A2* contribute to MED-4.

## Background

Multiple epiphyseal dysplasia (MED) is a heterogeneous genetic condition characterized by variable phenotypes, such as short stature (mild to moderate), joint deformities, abnormal gait, and early-onset osteoarthritis [1]. MED is associated with structural anomalies in epiphyses and delayed ossification of the epiphyses with small, irregular ossification centers, resulting in moderate shortening. Patients usually appear normal at birth and have good muscular development and normal intelligence [2].

Mutations in the following six genes, *COMP* (MIM 600310) [3], *COL9A1* (MIM 120210) [4], *COL9A2* (MIM 120260) [5], *COL9A3* (MIM 120270) [6], *MATN3* (MIM 602109) [7], and solute carrier family 26 member 2 (*SLC26A2*, MIM 606718) [8], have been found to be related to MED. *SLC26A2* exhibits autosomal recessive inheritance, but the other five genes show an autosomal dominant manner. Mutations in the *SLC26A2* result in a family of skeletal dysplasias depending on the residual sulfate transporter activity, which range in severity from the very severe achondrogenesis type IB (MIM 600972) [9], atelosteogenesis type II (MIM 256050) [10], and diastrophic dysplasia (MIM 222600) [11] to the relatively mild recessive multiple epiphyseal dysplasia-4 (MED-4, MIM 226900). The homozygous mutation c.835C > T (p.Arg279Trp) is the most common mutation in the *SLC26A2* gene, resulting in MED-4 with short stature, multiple epiphyseal dysplasia, scoliosis, double layered patella, brachydactyly and clubfoot [8, 12, 13].

We present two siblings with MED-4 from an eastern Chinese family. Genetic analysis revealed compound heterozygotes for two novel heterozygous mutations in *SLC26A2*. Further genetic studies and clinical evaluation of their parents revealed that these two mutations were from their father and mother, respectively. This study reported that compound heterozygous mutations in *SLC26A2* contributed to MED-4.

## Case presentation

### Patient 1

#### Clinical findings

A 12-year-old girl was born normally to non-consanguineous, healthy eastern Chinese parents after a normal pregnancy. The body length at birth was 51 cm. She was referred to our hospital for diagnosis and treatment. There was no family history of endocrine diseases and musculoskeletal problems. Her parents noticed unequal leg lengths around the age of 6 years and radiography revealed coxa plana. Abnormal gait and limping were noticed at 9 years. The rotation function of the right leg was limited. For these reasons, she underwent right hip arthroplasty and resection of cartilago acetabularis in another hospital. Postoperative pathology revealed chronic synovitis. Half a year after the surgery, a lump with high skin temperature was noticed by her parents in the left femoribus internus. The flexion-extension function of the left leg was limited. Physical examination revealed the following: height 138 cm. Her intellectual development and hearing were normal. She had brachydactyly, bilateral skewfoot, and lumbosacral scoliosis. The movements of both hips were limited. She did not have a cleft palate, cephalofacial deformities, or respiratory insufficiency. Routine analysis for common skeletal dysplasia excluded any thyroid or growth hormone disorders and immunopathies. Upon analysis, bone metabolism appeared normal.

#### Radiological findings

**Fig. 1:**
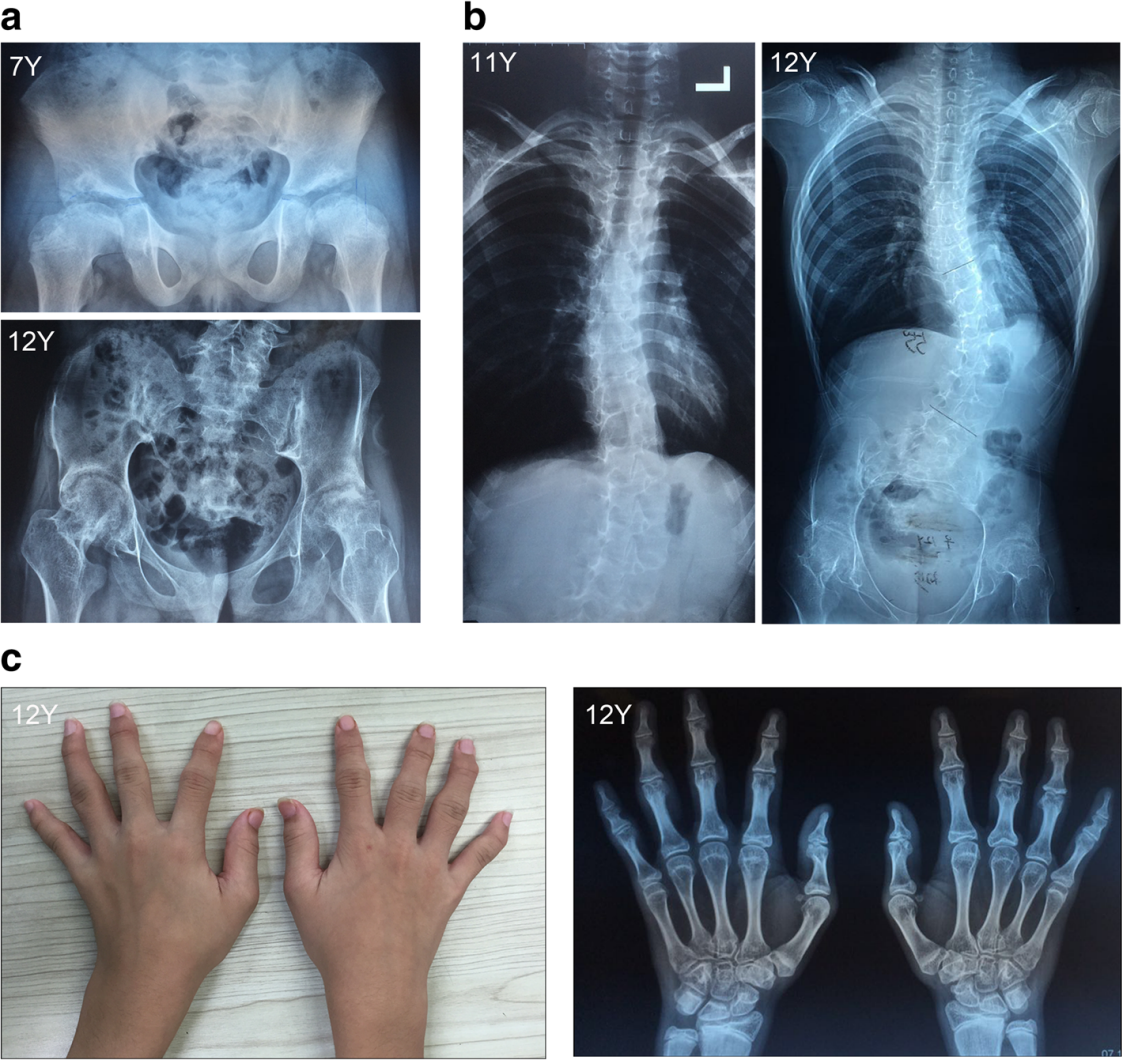
Radiographs and clinical features of patient 1. Hip radiographs revealed coxa plana at the ages of 7 and 12 years (**a**). Spinal radiographs confirmed the progress of scoliosis (**b**). Hand radiographs confirmed the brachydactyly and significantly flattened articular surface (**c**–**d**)

Radiological documentation at the ages of 7 and 12 years revealed hip dysplasia with the following deformities: short femoral necks, flattened and irregular femoral heads, and early closure of epiphysis (Fig. 1a). Spinal radiographs at the ages of 11 and 12 years confirmed evolving scoliosis, which appeared to be structural vertebral deformity (Fig. 1b). Hand radiographs confirmed the brachydactyly and significantly flattened articular surface. The metacarpi and phalanges were mild shorten (Fig. 1c).

### Patient 2

#### Clinical findings

A 6-year-old boy, the younger brother of patient 1, was also born to the same parents. The pregnancy was normal, and he was normal at birth without cleft palate or cephalofacial deformities. The body length at birth was 50 cm. An abnormal gait, waddling with short steps, was also noticed recently.

#### Radiological findings

**Fig. 2:**
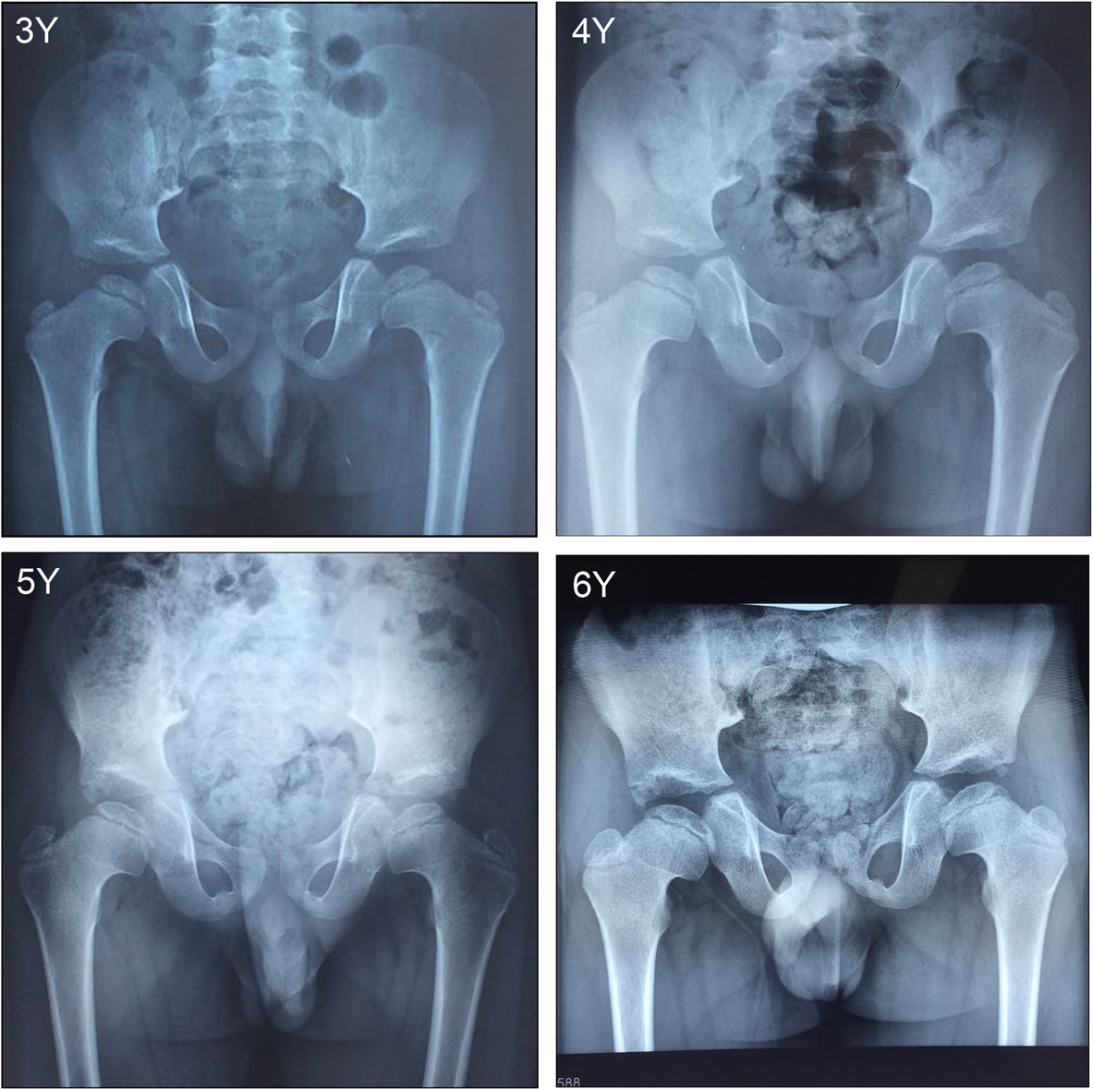
Hip radiographs of patient 2 during the ages from 3 to 6 years. Roentgenologic bone survey showed hip dysplasia with the following abnormalities: both femoral necks were short with flattened heads, acetabulum dysplasia, and secondary ossification center of femur dysplasia

Roentgenologic bone survey showed hip dysplasia with the following abnormalities: both femoral necks were short with flattened heads; acetabulum dysplasia, and secondary ossification center of femur dysplasia (Fig. 2).

### Molecular data

**Fig. 3:**
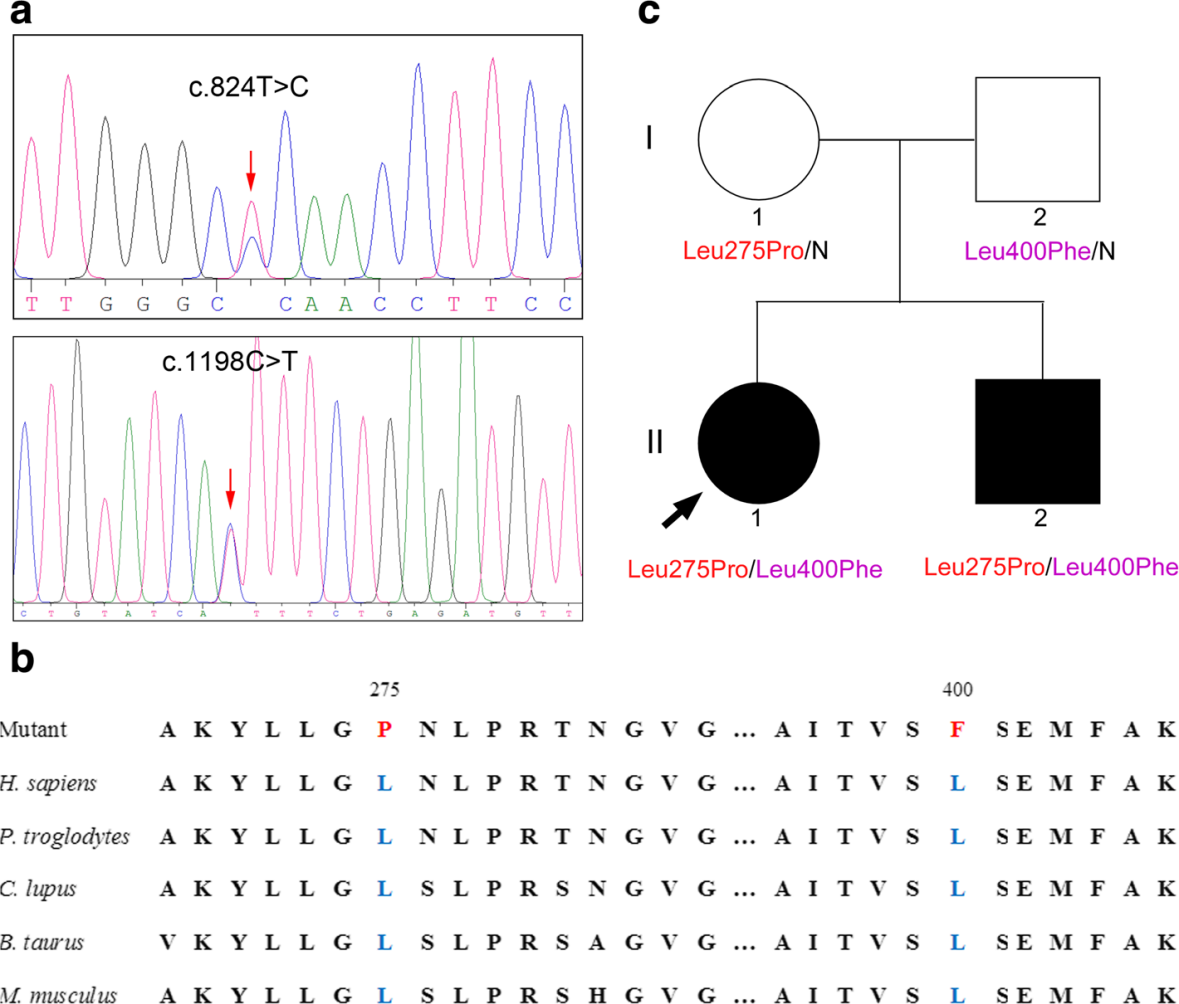
The missense mutations in *SLC26A2* in patient 1 and family pedigree. Sanger sequencing results revealed novel compound heterozygous mutations (**a**) in *SLC26A2* contributing to MED-4 (**c**). Both of these mutations are evolutionarily conserved (**b**)

Written informed consent was obtained from the parents of patient 1 and patient 2. Genomic DNA was extracted from peripheral blood using an e.Z.N.A.® Blood DNA Kit (Omega Bio-tek, Norcross, GA, USA) according to the manufacturer’s protocol. A total of 363 genes, including *COL1A1*, *COL1A2*, *COL11A1*, and other related genes, were analyzed by Targeted NGS in patient 1. The total size of the target regions of the capture array was 3.0 Mb. After filtering out common variants and neutral or benign mutations (allele frequency of ≥0.5% in dbSNP (http://www.ncbi.nlm.nih.gov/projects/SNP/), the 1000 Genomes Pilot Project Data (http://www.1000genomes.org/) or BGI in-house database, which includes 1092 normal subjects.), two mutations, *SLC26A2* c.824 T > C (NM_000112.3) and *SLC26A2* c.1198C > T were identified (Fig. 3a). Both variants were absent from all databases, including 1000genomes, dbsnp, ESP6500, and the BGI in-house database, and both variants were predicted as functional damaging in MutationTaster, Polyphen-2 and SIFT (Table 1). Both of the two mutations are evolutionarily conserved (Fig. 3b). Then sanger sequencing revealed two variants in patient 2; *SLC26A2* c.824 T > C and *SLC26A2* c.1198C > T were identified in Mother and Father, respectively (Fig. 3c).

**Table 1:** Functional prediction of *SLC26A2* mutations

Chr.	Position	Gene symbol	Transcript variant	Protein variant	MutationTaster score	PolyPhen-2	SIFT score
5	149359980	*SLC26A2*	c.824 T > C	p.Leu275Pro	0.999	0.999	0.000
5	149360354	*SLC26A2*	c.1198C > T	p.Leu400Phe	0.999	1.000	0.002

## Discussion and conclusion

Multiple epiphyseal dysplasia is a heterogeneous group of skeletal dysplasias characterized by dysplastic epiphyses at multiple sites [14]. Superti-Furga et al. first reported a homozygous *SLC26A2* mutation (c.835C > T, p.Arg279Trp) in a 36-year-old man of tall-normal stature with MED-4 [12]. Variable phenotype with variable joint manifestations and normal to short stature were described in 18 individuals with MED-4 [8]. The deformity of clubfoot was observed in approximately 28% of the MED-4 patients. The most frequent radiographic finding was mild to moderate hip dysplasia. Only one patient had undergone hip replacement surgery for hip dysplasia. That patient required varisation osteotomies of both femoral necks. Other characteristic findings included brachydactyly and scoliosis [14]. A double-layer patella seems to be specific but not essential to MED-4 and when present, separates the condition from the dominant forms of MED caused by mutations in *COMP*, *COL9*, and *Matrilin-3*.

In the present study, the presence of short stature, coxa plana, brachydactyly, abnormal gait, and scoliosis in Patient 1 led to the clinical diagnosis of MED-4. Very recently, the abnormal gait and coxa plana were noticed in the younger brother, Patient 2, at the same age as his elder sister did at 6 years. To verify the cause of the disease, we suggested their parents to authorize a genetic analysis using an available capture array, which covers 363 genes related to bone diseases, including *COL1A1*, *COL1A2*, *COL11A1*, *SLC26A2*, *COMP*, *COL9A1*, and other genes. Indeed, the younger brother harbored the same mutations in *SLC26A2*. We then suspected him to be at the early stage of MED-4, and we advised his parents to be cautious regarding the development of epiphyseal dysplasia in the future.

Mutations in *SLC26A2* are related to a wide range of phenotypes, depending on the residual sulfate transporter activity. These phenotypes range in severity from the very severe achondrogenesis type IB, atelosteogenesis type II, and diastrophic dysplasia to the relatively mild recessive MED-4. The most common *SLC26A2* mutation reported in several studies is homozygous c.835C > T (p.Arg279Trp) [15]. Karniski compared the sulfate transport activity of 11 SLC26A2 mutations in the *Xenopus laevis* oocyte expression system [16]. Their results indicated that the p.Arg279Trp mutation transported sulfate at a rate 32% that of wild-type SLC26A2, while some mutations had minimal residual sulfate transport function. Makitie et al. reported another homozygous *SLC26A2* mutation, c.1957 T > A (p.Cys653Ser), in two unrelated patients with hip dysplasia, recurrent patella dislocation, and normal stature [14]. Very recently, a patient from a Caucasian three-generational family with MED-4 was reported to be a compound heterozygote for the common mutation in *SLC26A2* and a novel mutation, p.Ser522Phe, while her maternal grandfather was homozygous for the common mutation [15]. Using a skeletal dysplasia targeted NGS panel, two novel heterozygous *SLC26A2* mutations were identified in Patient 1. These mutations, c.824 T > C (p.Leu275Pro) and c.1198C > T (p.Leu400Phe), are located in the extracellular loop (between amino acids 263 and 296) and the cytoplasmic loop (between amino acids 399 and 420), respectively. Both mutations were predicted to be functionally deleterious and missing in the above-mentioned databases. However, no functional studies have been undertaken for both mutations, so we do not know exactly if these are severe or mild mutations. Sanger sequencing confirmed that these mutations in Patients 1 and 2 came from their parents, one mutation from each. Clinical features and genetic analysis suggested that in this Eastern Chinese family, both patients were also compound heterozygotes for two novel *SLC26A2* mutations.

In conclusion, we present two patients of MED-4 with evolving clinical and radiological features. Skeletal surveys, joint complications, and genetic testing of their parents were found to be essential to understanding the mechanism. Both patients were compound heterozygotes for two unreported mutations in *SLC26A2*, c.824 T > C (p.Leu275Pro) and c.1198C > T (p.Leu400Phe).

## Abbreviations

DNA: Deoxyribonucleic acid
MED: Multiple epiphyseal dysplasia
MED-4: Multiple epiphyseal dysplasia, type 4
MIM: Mendelian inheritance in man
SIFT: Sorting intolerant from tolerant
Targeted NGS: Targeted next-generation sequencing
